# Comprehensive analysis of KLF2 as a prognostic biomarker associated with fibrosis and immune infiltration in advanced hepatocellular carcinoma

**DOI:** 10.1186/s12859-023-05391-0

**Published:** 2023-06-29

**Authors:** Xue-Qin Chen, Jie Ma, Di Xu, Zuo-Lin Xiang

**Affiliations:** 1 Department of Radiation Oncology, Shanghai East Hospital, School of Medicine, Tongji University, Shanghai, 200120 China; 2 Department of Radiation Oncology, Shanghai East Hospital Ji’an hospital, Jiangxi, 343000 China

**Keywords:** Hepatocellular carcinoma, KLF2, Prognosis, Fibrosis, Immune infiltration

## Abstract

**Purpose:**

Most Hepatocellular carcinoma (HCC) patients are in advanced or metastatic stage at the time of diagnosis. Prognosis for advanced HCC patients is dismal. This study was based on our previous microarray results, and aimed to explore the promising diagnostic and prognostic markers for advanced HCC by focusing on the important function of KLF2.

**Methods:**

The Cancer Genome Atlas (TCGA), Cancer Genome Consortium database (ICGC), and the Gene Expression Comprehensive Database (GEO) provided the raw data of this study research. The cBioPortal platform, CeDR Atlas platform, and the Human Protein Atlas (HPA) website were applied to analyze the mutational landscape and single-cell sequencing data of KLF2. Basing on the results of single-cell sequencing analyses, we further explored the molecular mechanism of KLF2 regulation in the fibrosis and immune infiltration of HCC.

**Results:**

Decreased KLF2 expression was discovered to be mainly regulated by hypermethylation, and indicated a poor prognosis of HCC. Single-cell level expression analyses revealed KLF2 was highly expressed in immune cells and fibroblasts. The function enrichment analysis of KLF2 targets indicated the crucial association between KLF2 and tumor matrix. 33-genes related with cancer associated fibroblasts (CAFs) were collected to identify the significant association of KLF2 with fibrosis. And SPP1 was validated as a promising prognostic and diagnostic marker for advanced HCC patients. CXCR6 CD8^+^ T cells were noted as a predominant proportion in the immune microenvironment, and T cell receptor CD3D was discovered to be a potential therapeutic biomarker for HCC immunotherapy.

**Conclusion:**

This study identified that KLF2 is an important factor promoting HCC progression by affecting the fibrosis and immune infiltration, highlighting its great potential as a novel prognostic biomarker for advanced HCC.

**Supplementary Information:**

The online version contains supplementary material available at 10.1186/s12859-023-05391-0.

## Introduction

Hepatocellular carcinoma (HCC), one of the most common and invasive solid malignancies, accounts for the most proportion of liver cancers. The incidence rate of primary liver cancer ranks fifth and the mortality is the third highest, causing approximately 906,000 new cases and over 830,000 deaths per year [1]. Most HCC patients are in advanced or metastatic stage at the time of diagnosis. However, more desperately, the current clinical treatments for advanced HCC patients does not yield good results. Once the tumor has been advanced and metastasized, the HCC patient's prognosis is very bleak [2]. Lymph node is one the most frequent metastasis site for primary HCC. Accordingly, lymph node metastasis occurs in about half of HCC patients with extrahepatic metastasis, and these patients only have a median survival of less than 1 year [3]. Thus, understanding the mechanism of the advancement and metastasis of HCC is essential to propose new therapeutic strategies for patients. And it is imperative to exploit new diagnostic approaches and treatment strategies for advanced HCC patients.

Based on our previous microarray results [4], we identified several lncRNAs as important factors in HCC progress. Lnc-EPS15L1-2:1 is strongly associated with metastasis and advancement in HCC [5]. However, the molecular regulation of lnc-EPS15L1-2:1 in advanced HCC is still unclear. Accordingly, lncRNAs regulate both coding and noncoding genes via cis- and trans- regulatory signals [6, 7]. Surprisingly, we found that KLF2 was a predictive target gene of lnc-EPS15L1-2:1 from both cis- and trans- analysis, which implied a potential interaction between KLF2 and the progress of HCC.

KLF2, once termed lung Krüppel-like factor (LKLF), is a transcription factor from Krüppel-like factor family. The family genes, characterized by a zinc-finger-containing DNA binding domain, regulate downstream gene transcription via binding to GC-rich DNA sequences [8]. KLF2 is involved in many major biological processes, including proinflammatory activation, cell proliferation, apoptosis, and metabolism (such as glucose metabolism, fatty acid and cholesterol metabolism, amino acid and protein metabolism and so on) [9–13]. In addition, researches have reported that KLF2 is significantly dysregulated in many solid malignancies, including gastric cancer [14], non-small-cell lung cancer [15], pancreatic ductal adenocarcinoma and prognostic cancer [16, 17]. Moreover, KLF2 has been demonstrated to be a significantly terminal factor for tumor progress and metastasis in multiple cancers [18–20]. Therefore, based on previous results and existing studies, we speculated that KLF2 exert an important biological effect in the lnc-EPS15L1-2:1-related pathway axis to promote HCC progression.

Here, we focus on exploring how KLF2 influences biological changes to promote HCC progression. Interestingly, we found that KLF2 is highly expressed in fibroblasts and immune cells, but low in hepatocytes. Therefore, we further studied the association between cancer associated fibroblasts (CAFs) and KLF2. Results showed that the CAFs-related target gene, SPP1, is a key regulatory gene of KLF2 affecting cancer fibrosis, and is also a promising diagnostic factor for HCC. On the other hand, additionally, the important relationship between immune cells, especially T cells, and KLF2 was also analyzed. We found that an important T-cell receptor (TCR) molecule, CD3D, plays a significant role in KLF2 affecting the immune microenvironment. The results also showed that CD3D has a strong predictive role in HCC immunotherapy. In conclusion, our study provides a novel clue that KLF2 is a considerable contributor for advanced HCC by affecting the fibrosis and immune infiltration, providing new perspectives on exploring the molecular mechanism for HCC advancement, and emphasizing the potential of KLF2 for improving the prognosis of advanced HCC patients in clinical practice.

## Materials and methods

### Data source and processing

The gene expression profile was derived from The Cancer Genome Atlas (TCGA, https://portal.gdc.cancer.gov), the International Cancer Genome Consortium database (ICGC, https://daco.icgc.org/), and the Gene Expression Comprehensive Database (GEO, http://www.ncbi.nlm.nih.gov/geo). TCGA_LIHC and ICGC_LIRI transcriptome data were used for expression correlation analysis and prognostic analysis. GSE25097 and GSE6764 from GEO database were used to explore genes expression distribution. The sequencing data and clinical prognosis data of the HCC multi-cohorts were aggregated by the BEST tool (https://rookieutopia.com/) with batch effects removed to analyze the correlation of gene expression with drug susceptibility, immune infiltration, and prognosis of ICI treatment.

The HCC cohhort, Firehose Legacy cohort, from the cBioPortal platform (https://www.cbioportal.org/) was applied to analyze the mutational landscape and relevant correlation. Single-cell sequencing data were analyzed in CeDR Atlas platform (https://ngdc.cncb.ac.cn/cedr/) and the Human Protein Atlas (HPA, https://www.proteinatlas.org/). The identified KLF2 transcription factor target gene sets were collected from Harmonizome platform (https://maayanlab.cloud/Harmonizome/) to go further analyzing.

For unnormalized RNA-seq data, the raw expression values of genes were log2 transformed. If a gene is traced to multiple probes, the average expression value was taken to representative gene expression levels when using microarray data from the GEO database. Probe entries mapping to unrecorded gene IDs or multiple gene IDs was eliminated. Finally, according to the annotation of the corresponding microarray platform, probe IDs were converted to gene symbols. For the overall expression level of the gene signature, the single sample Gene Set Enrichment Analysis (ssGSEA) algorithm is applied to evaluate the gene enrichment fraction in each sample, thus differentiating high and low expression groups of the gene signature.

### Analysis of KLF2 methylation and m6A correlation

N6-methyladenosine (m6A) is an RNA modification that involves the addition of a methyl group to the nitrogen atom at the sixth position (N6) of adenosine. This modification plays a critical role in various biological processes, including mRNA splicing, translation, stability, and degradation. Moreover, it has been implicated in numerous physiological and pathological processes, such as cancer, immune responses, and viral infection [21, 22]. To explore comprehensively the methylation of KLF2 promoter, we used the deoxyribonucleic acid (DNA) methylation data from the online MethSurv tool (https://biit.cs.ut.ee/methsurv/) to analyze different methylation sites of KLF2 and the survival data in TCGA cohort of HCC. The m6A-related genes were derived from the research by Juan Xu et al. on the molecular characterization and clinical significance of m6A modulators across 33 cancer types [23].

### Analysis of differential expression genes and functional enrichment

Limma package in the R software was used to study the differentially expressed mRNA. The threshold was defined as “adjusted *P* < 0.05 and log2 (Fold Change) > 1 or log2 (Fold Change) <  − 1” for the differential expression of mRNAs. For functional enrichment, Gene Ontology (GO), a widely-used tool, is utilized to annotate genes with functions, especially molecular function, biological pathways, and cellular components, and Kyoto Encyclopedia of Genes and Genomes (KEGG) enrichment analysis [24], a practical resource, is used to study gene functions and associated high-level genome functional information. ClusterProfiler package (version: 3.18.0) in R was employed to analyze the GO function of potential targets and enrich the KEGG pathway. The R software ggplot2 package was used to draw boxplot and the pheatmap package was used to draw heatmap.

### Analysis of immune infiltration and Kaplan–Meier survival

To assess the immune score evaluation, we used immuneeconv, an R software package integrating six latest algorithms, including TIMER, xCell, MCP-counter, CIBERSORT, EPIC and quanTIseq. The immune scoring results were displayed visually through R software package “ggplot2” and “pheatmap”. Kaplan–Meier survival curves were based on RNA sequencing data and corresponding clinical information, which were analyzed and visualized by the “survival” and “surviviner” R packages.

### Subtype grouping

Based on the RNA sequencing data and corresponding clinical information of 371 HCC samples in the TCGA dataset, consistency clustering was performed using the R software package “ConsensesclusterPlus” (v1.54.0), and the parameters were set as follows: the maximum number of clusters was 6, 80% of the total samples were extracted 100 times, clusterAlg = “HC”, innerlinkage = “ward”, D2'. The cluster heatmap was visualized using the “pheatmap” (v1.0.12) R package. Gene expression heatmap was drawn by the “survival” and “surviviner” R packages, and genes with a variance greater than 0.1 were retained.

### Screening of prognostic factors and establishment of nomogram

The “forestplot” R package was used to construct the forest map after performed multivariate cox regression analysis. A nomogram was developed based on the results of multivariate cox proportional hazards analysis to predict the 1-, 3-, and 5-year overall recurrence. And the nomogram was established through “rms” R package.

## Results

### Decreased KLF2 expression is associated with poor survival outcome

**Fig. 1:**
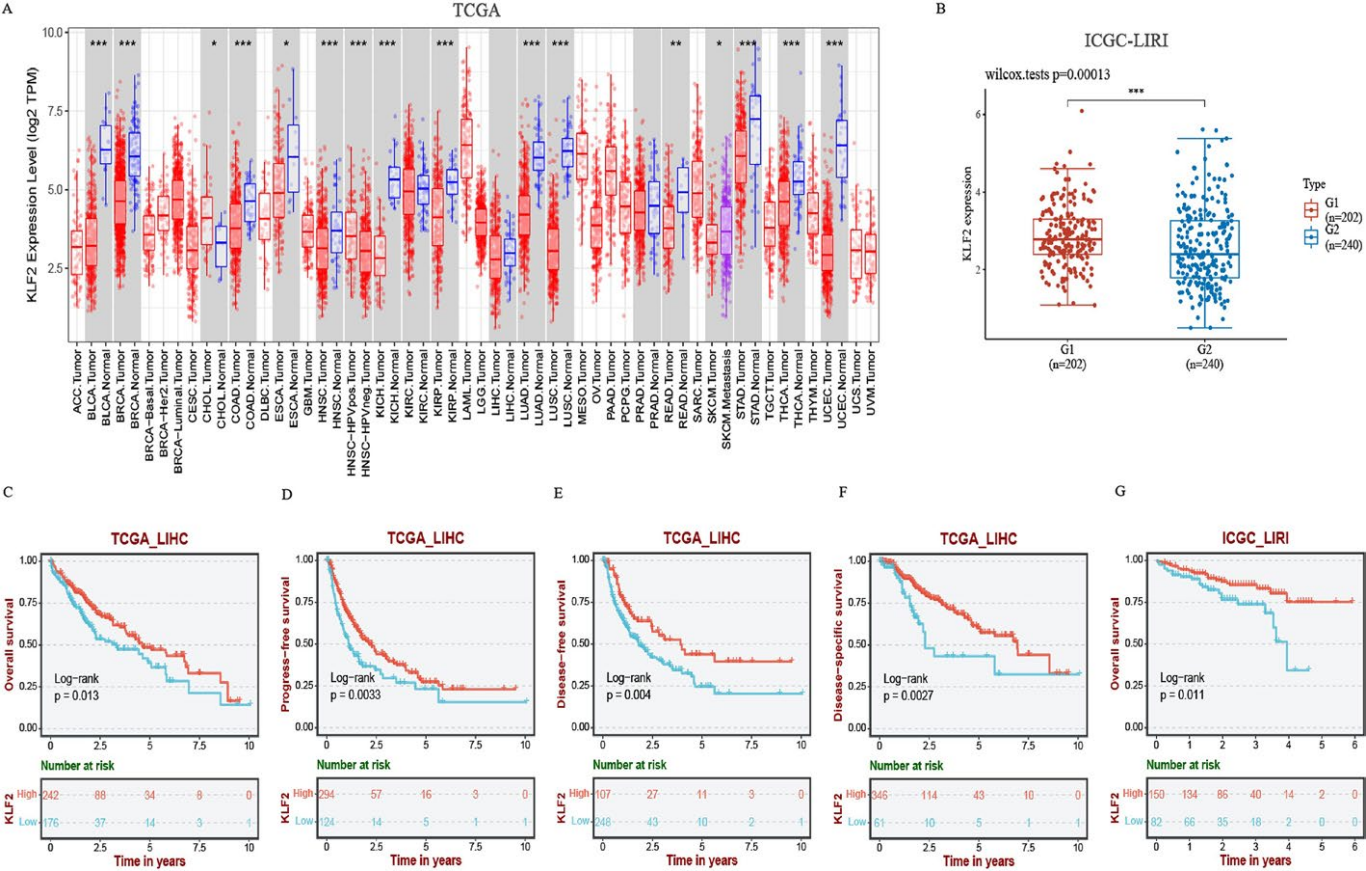
The expression distribution and prognostic survival value of KLF2 in tumor tissues and normal tissues of TCGA-LIHC and ICGC-LIRI. **A** The distribution of KLF2 expression across different types of tumor and normal tissues. **B** The level of KLF2 expression was significantly lower in tumor tissue in ICGC-LIRI (G1: normal liver tissue; G2: primary tumor tissue). **C**–**F** Kaplan–Meier survival analysis showed patients with higher KLF2 expression had a significantly better overall survival (OS), progression free survival (PFS), disease free survival (DFS) and disease specific survival (DSS) from TCGA-LIHC. **G** Kaplan–Meier survival analysis of KLF2 from ICGC-LIRI dataset. *P < 0.05, **P < 0.01, ***P < 0.001

Firstly, we analyzed KLF2 expression distribution between different human tumor and normal tissues in TCGA database. The result presented that KLF2 expression is significantly down-regulated in multiple solid cancers (Fig. 1A), such as breast invasive carcinoma (BRCA), colon adenocarcinoma (COAD), kidney renal papillary cell carcinoma (KIRP), lung adenocarcinoma (LUAD), lung squamous cell carcinoma (LUSC), rectum adenocarcinoma (READ) and so on. Additionally, Fig. 1B showed a significant down-regulation of KLF2 expression in the ICGC_LIRI cohort.

In addition, as a supplement, we also explore the expression distribution and prognostic value of several KLF family members (including zinc finger transcription factors) and regulators of NOS enzymes, including KLF2, KLF4, KLF5, KLF6, KLF8, KLF9, KLF10, KLF11, KLF12, NOS2 and NOS3 [25–28]. The expression distribution of these genes in TCGA database and LIRI databases was initially examined. The results indicated that, as depicted in the Additional file 1: Fig. S1, KLF5 and KLF12 did not exhibit significant differential expression in HCC in either database.

Then survival prognosis of the analyzed genes was assessed using Kaplan–Meier curves in TCGA_LIHC and ICGC- LIRI. Results showed that KLF2 was the only gene found to have a significant correlation with overall survival (OS), progression free survival (PFS), disease free survival (DSS), and disease specific survival (DSS). This means that HCC patients with higher level of KLF2 expression had a significantly better OS, PFS, DFS, and DSS (Fig. 1C–G, Additional file 1: Fig. S2).

### Analysis of gene expression regulation of KLF2 in HCC from genomic alteration landscape and methylation modifications

**Fig. 2:**
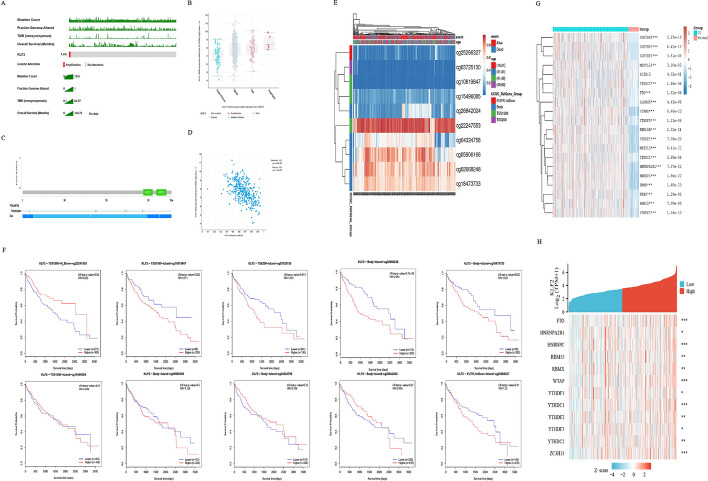
An overview of KLF2 genetic alterations landscape in HCC. **A** The presentation of KLF2 mutation landscape in HCC. **B** Putative copy number alterations of KLF2 in HCC. **C** Visualization of KLF2 genetic landscape in HCC. **D** The correlation between KLF2 methylation and the mRNA expression level. **E** The heatmap of the methylation level of KLF2 in HCC and normal samples. **F** The Kaplan–Meier survival curves of different islands of KLF2 in HCC. **G** The expression distribution of m6A-related genes in HCC and normal tissues. **H** The correlation between KLF2 expression and m6A-related genes in HCC. *P < 0.05, **P < 0.01, ***P < 0.001

To analyze the expression regulation of KLF2 in HCC comprehensively, we used cBioPortal platform to investigate the genetic mutation status of KLF2. As shown in Fig. 2A, B, KLF2 genomics presented a hypo-mutation condition in most of HCC patients in Firehose Legacy cohort. Only a small percent (1.4%) patients were accompanied by the KLF2 amplification of copy number variation (CNV). The sequence features of KLF2 presented that there were highly conserved classical Cys2/His2 zinc fingers (Fig. 2C). Furthermore, Fig. 2D showed KLF2 expression was negatively correlated with its methylation (Spearman =  − 0.57, *P* < 0.001).

Then, we used Methsurv platform to further analyze methylation level of KLF2 in HCC. The heatmap result showed 10 CpG islands of KLF2 and the corresponding methylation levels. We observed that cg22247553 had the highest level of DNA hypermethylation (Fig. 2E). Besides, we noticed that the methylation level of five CpG sites, cg22247553, cg10819847, cg03725130, cg02668248, and cg18473733, were associated with prognosis (Fig. 2F). And patients with KLF2 hypermethylation of cg22247553, had a worse survival prognosis. Afterwards, we assessed the variance of the expression levels of the m6A-related genes between HCC cancer and normal tissues. As expected, a significantly higher percentage of m6A genes expression was detected in cancer tissues (Fig. 2G). Furthermore, Fig. 2H demonstrated there was a strong linkage between KLF2 exposure and m6A-related genes. These foregoing results indicate that the low expression level of KLF2 in HCC mainly is regulated by hypermethylation, instead of genetic mutation.

### Single-cell level expression analysis reveals a high KLF2 expression in fibroblasts and immune cells, while low expression in hepatocytes

**Fig. 3:**
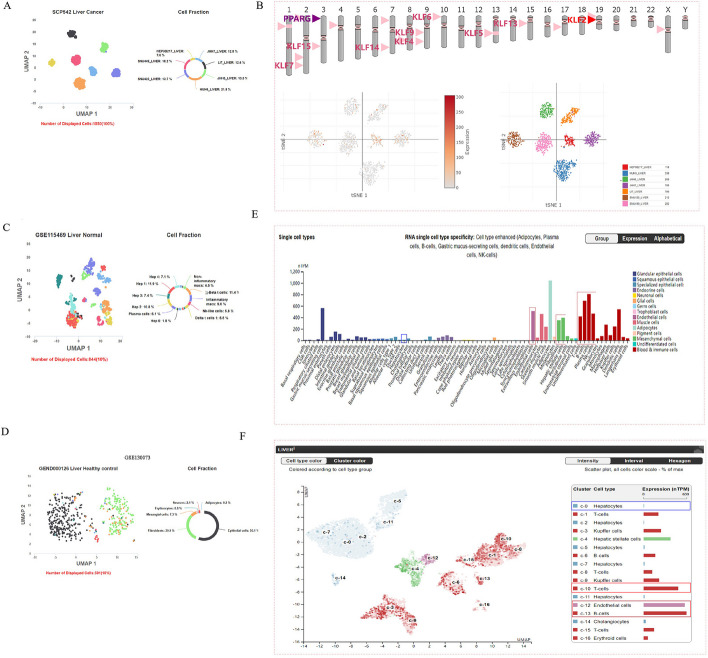
A single-cell level expression analysis of KLF2 in liver tissue. **A** UMAP plot and cell fraction plot show the cell line type clustering and cell line ratio of liver cancer based on the single-cell sequencing analysis from the SCP542. **B**, **C** UMAP plot and Cell Fraction plot show the cell type clustering and cell ratio in the normal liver tissue based on the single-cell sequencing analysis from the GSE 115469 and the GSE130073. **D** TSNE plot shows KLF2 expression in different cell lines of liver cancer. **E**, **F** Box bars show the KLF2 expression in different cell types at single cell level in normal liver tissue

To analyze KLF2 expression distribution in different cell types in liver cancer and normal tissue, we used Uniform Manifold Approximation and Projection (UMAP) and t-distributed Stochastic Neighbor Embedding (tSNE) algorithm for the single cell expression analysis in multiple platforms. Figure 3A and B showed the single-cell expression level of KLF2 in different cell lines of the SCP542 analysis [29] in the Cellular Drug Response (CeDR) Atlas and Single cell portal (SCP) database. Using the CeDR Atlas, we further analyzed the single-cell sequencing data of hepatic cells derived from GSE115469 [30] and GSE130073 [31]. The UMAP and the Cell Fraction plots showed the epithelial cells and fibroblasts make up a substantial proportion among all liver cells, which together accounted for nearly 90% (Fig. 3C, D). Then we used HPA platform to further analyze the single-cell expression level of KLF2 in liver tissues. The bar graph results exhibited that KLF2 was mostly expressed in fibroblasts, epithelial cells and immune cells (T-cells, B-cells, plasma cell, NK-cells and so on) (Fig. 3E, F). Surprisingly, we noticed a low KLF2 expression in hepatocytes. These results suggest that KLF2 expression levels are significantly different in different cell types in the liver tissue, mainly focusing on fibroblasts, epithelial cells and immune cells, which are the main components of liver tissue.

### Functional enrichment analysis of KLFTs

**Fig. 4:**
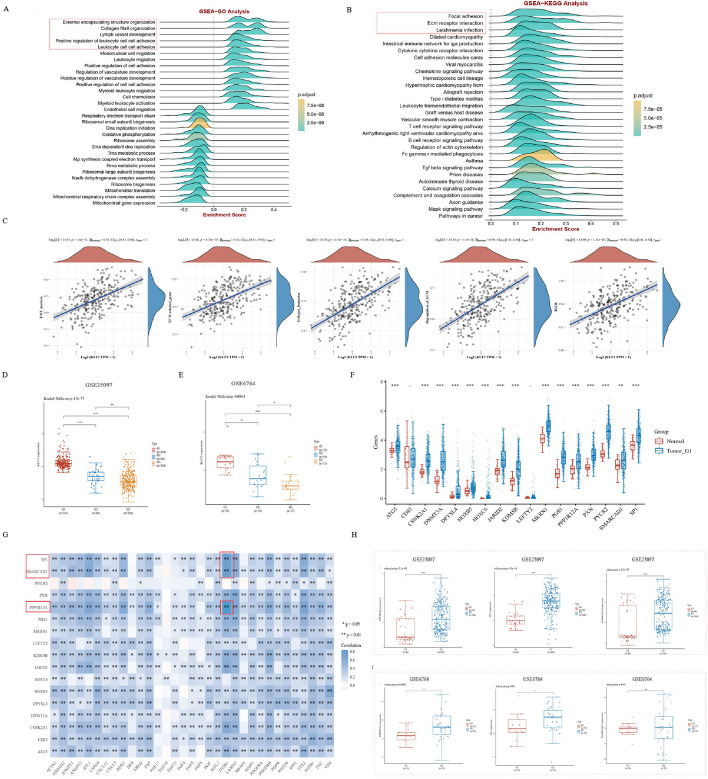
Analysis of KLF2 targets (KLFTs) and KLF2 expression in different cohorts. **A**, **B** GSEA-GO analysis and GSEA-KEGG analysis [24] on the 98 targets of the transcription factor KLF2. **C** The correlations between KLF2 and relevant pathway score. **D** KLF2 expression is analyzed in different tissues from GSE25097. G1: normal liver tissue; G2: liver cirrhosis tissue; G3: HCC tissue. **E** KLF2 expression is analyzed in different tissues from GSE6764. G1: liver cirrhosis tissue; G2: early HCC tissue; G3: advanced HCC tissue. **F** The expression distribution of KLFTs-17 in the normal and HCC samples from TCGA. **G** The correlations between KLFTs-17 and CAFs marked genes. The expression levels of PPP1R12A, SP1 and SMARCAD1 are analyzed in the GSE25097 (**H**) and GSE6764 (**I**) (G1: liver cirrhosis tissue; G2: HCC tissue). *P < 0.05, **P < 0.01, ***P < 0.001

KLF2 is an important transcription factor from the Krüppel-like family of proteins, which exerts regulation in various cell types in the processes of activation, differentiation and migration by targeting different downstream genes. We collected 98 target genes of the KLF2 from the CHEA Transcription Factor Targets dataset in Harmonizome platform, namely KLFTs (Additional file 2: Table S1). The single sample Gene Set Enrichment Analysis (ssGSEA) was used to calculate absolute enrichment scores, and Gene Set Enrichment Analysis (GSEA) was used to demonstrate the biological processes. We observed that the top several enrichment entries were concentrated in the biological characteristics related to tumor matrix and immunity. For GO analysis shown in Fig. 4A, the major items were “External encapsulating structure organization”, “Collagen fibril organization”, “Lymph vessel development”. For KEGG analysis in Fig. 4B, enriched items mainly were “Focal adhesion”, “ECM receptor adhesion”, “Leishmania infection”. Furthermore, we examined the correlation between KLF2 and common signaling pathways associated with tumor matrix formation. As we expected, KLF2 was in a high positive correlation with these pathways, such as EMT_markers, ECM_related genes, collagen_ formation, degradation_of_ECM and TGF β (Fig. 4C). EMT is primarily executed by EMT-activating transcription factors that belong to families such as SNAIL, TWIST, and ZEB and so on [32, 33]. Subsequently, a correlation analysis was conducted between KLF2 and EMT-markers, such as SNAI1, SNAI2, TWIST1, TWIST2, ZEB1, ZEB2, VIM and ID1. As depicted in Additional file 1: Fig. S3A–H, the study findings revealed a positive association between KLF2 expression levels and these EMT markers.

Fibroblasts account for the major stromal cell type in the microenvironment of liver diseases, including liver cirrhosis and liver cancers. Cirrhosis is a predominant contributor to the development of HCC. Therefore, we further analyzed the distribution of KLF2 expression in two datasets related with cirrhosis development, GSE 25097 and GSE 6764, to investigate the different expression of KLF2 in the tumor tissue compared to cirrhosis tissue. In Fig. 4D, the results analyzed from GSE 25097 showed KLF2 expression level continued to decreasing, as liver tissue became progressively cirrhotic and then progressed to HCC (G1: normal liver tissue; G2: liver cirrhosis tissue; G3: HCC tissue). The identical results were also observed in GSE 6764 in Fig. 4E (G1: liver cirrhosis tissue; G2: early HCC tissue; G3: advanced HCC tissue). In addition, we examined the expression distribution of SNAI1, ZEB2, and VIM, which are the top three markers exhibiting strong correlation with KLF2, in the GSE25097 dataset related to cirrhosis development. The findings revealed that normal tissues exhibited higher expression levels of SNAI1, ZEB2, and VIM compared to cancer tissues, and cirrhotic tissues showed higher expression levels than cancerous tissues as well (Additional file 1: Fig. S3I–K). The above results suggest that KLF2 is involved in the regulation of biological processes associated with tumor matrix. These results further demonstrate an essential role that KLF2 performed during development of HCC associated with liver fibrosis/cirrhosis.

### Identification a prognostic signature based on the KLFTs

In order to identify the key target genes of KLF2 in HCC, we successively performed the co-expression analysis and univariate Cox regression analysis of KLFTs (Additional file 3: Table S2, Additional file 1: Fig. S4). Then we obtained 17 prognostic significant genes, named KLFTs-17. Figure 4F showed that these 17 targets were over-expressed in HCC tumor tissues compared to the normal tissues.

Fibrosis of the tumor stroma is an important biological process contributing to the advancement of solid tumors. And cancer associated fibroblasts (CAFs) are the primary cells involved in this process. An increasing number of studies have reported that CAFs play a crucial role in promoting solid tumorigenesis. To further investigate the association between KLFTs and CAFs, we first collected 33 common CAFs-related marker genes by consulting relevant references [34–37] (Additional file 4: Table S3). Figure 4G showed the strong association of KLF2 with CAFs-related marker genes. Furthermore, we selected three KLF2 target genes (PPP1R12A, SP1 and SMARCAD1) with the strongest association with CAFs-related genes among KLFTs for expression level analysis in the GSE25097 and GSE6764 (Fig. 4H, I). Obviously, the expressions of PPP1R12A, SP1 and SMARCAD1 in HCC tissues were higher than those in the liver cirrhosis tissues, which were opposite to the expression trend of KLF2 in HCC. The above results suggest that KLF2 negatively regulates the expression of those downstream genes associated with fibrosis.

### Multidimensional analysis of subgroups classified by KLFTs-17

**Fig. 5:**
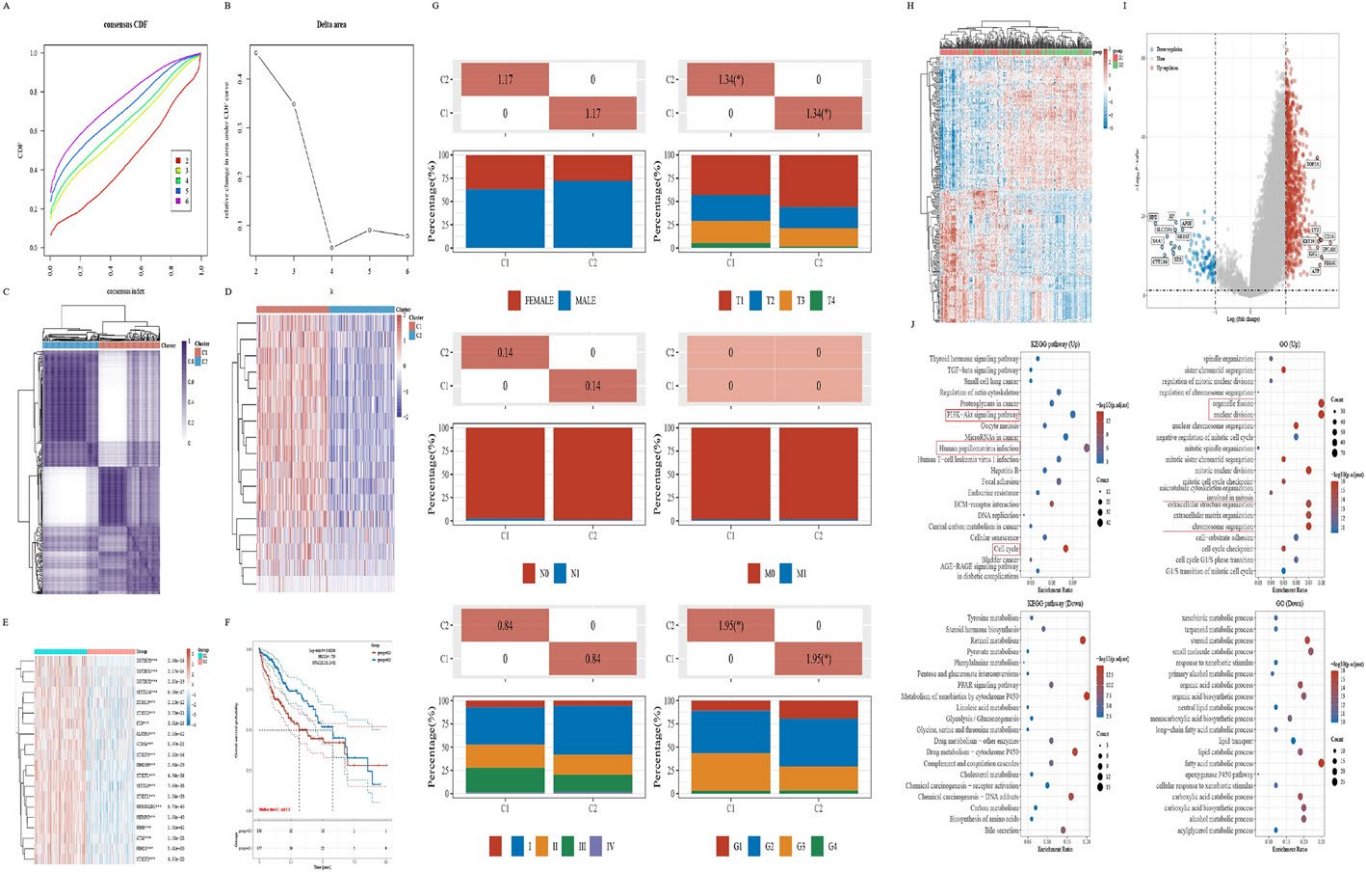
Subtype classification based on KLFTs-17 s. **A** Cumulative distribution function (CDF) based on consensus clustering. **B** Relative change in the area under the CDF curve (CDF delta area). **C** Heat map of consistent clustering results when k = 2, rows and columns represent samples, and different colors represent different subtype groups. **D** Heat map of KLF2-target genes expression between the two subgroups. **E** The expression distribution of m6A-related marker genes between the two subgroups. **F** Kaplan–Meier survival analysis of the two subgroups. **G** The distribution of clinical characteristics in samples of different subgroups, in which the horizontal axis represents different groups, the vertical axis represents the percentage of clinical information contained in corresponding grouped samples, and different colors represent different clinical information; The above table represents the distribution of a clinical feature in two groups (*P < 0.05). **H** The heatmap of the differential gene expression between the two subgroups. **I** The volcano plot was constructed using the fold change values and *P* adjust. Red dots indicate upregulated genes; blue dots indicate downregulated genes; grey dots indicate not significant. **J** Enrichment results, the enriched KEGG signaling pathways [24] and Gene Ontology (GO) analysis, of the differential genes expression between the two subgroups

We used cumulative distribution function (CDF) based on consensus clustering and classfied TCGA_LIHC samples into two subtype groups with k = 2 selected as the optimum (Fig. 5A–C). The cluster heatmap in Fig. 5D showed that the expression level of genes of the subgroup C1 were higher than that in the subgroup C2. Then we explored the expression distribution of m6A-related marker genes between subgroups. The results showed that the gene expression level differed distinctly between subgroups, and the expression level was higher in C1 subtype (Fig. 5E).

**Fig. 6:**
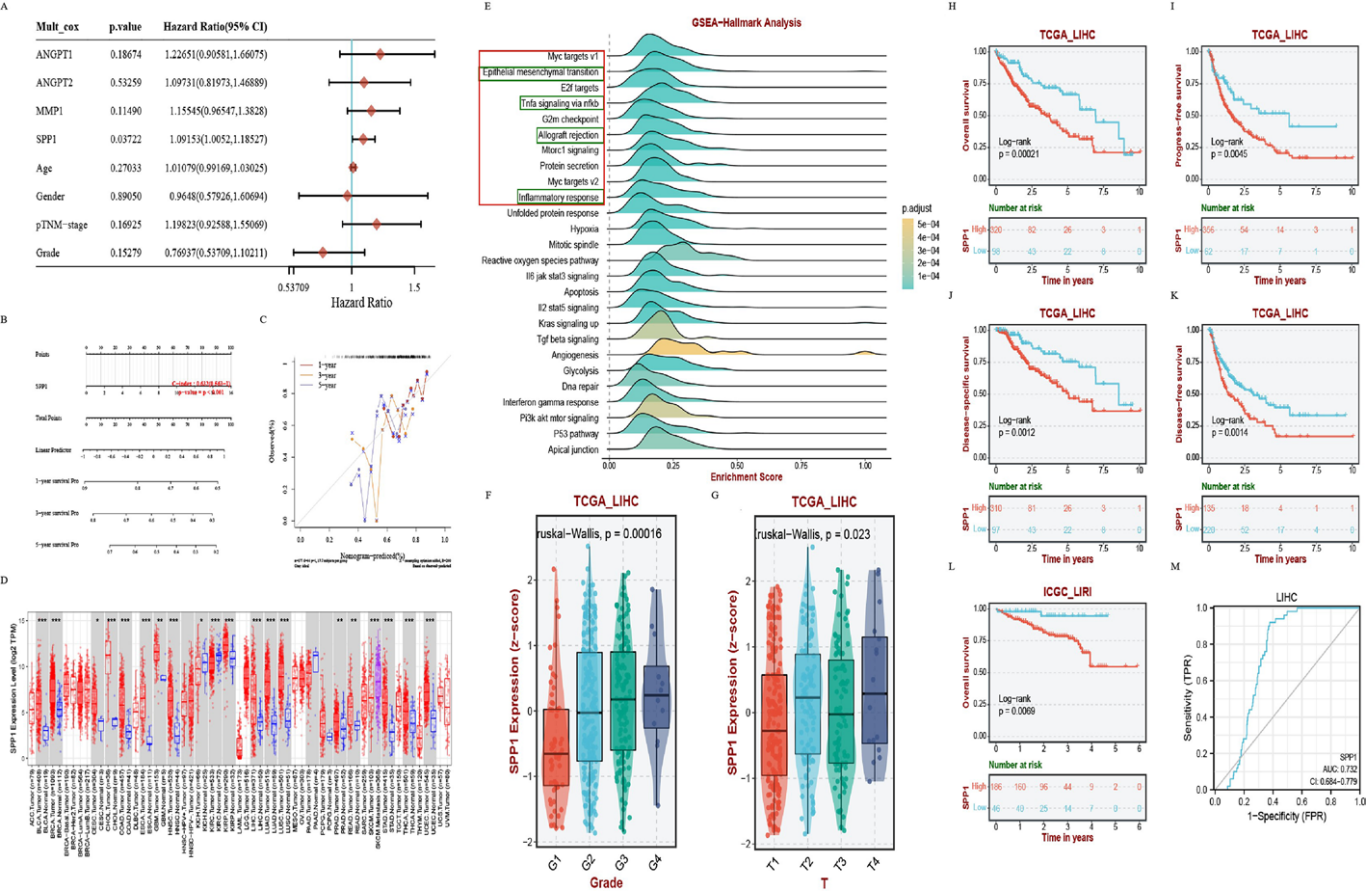
Confirmation of the prognostic factor SPP1. **A** Multivariate Cox regression among CAFs-related genes followed by multivariate Cox regression in the subgroup C1. **B** Nomogram showing the prediction of the 1-year, 2-year, and 3-year overall survival of HCC cancer patients. **C** Calibration curve for the overall survival nomogram model in the discovery group. **D** The distribution of SPP1 expression across different types of tumor and normal tissues. **E** GSEA-Hallmark analysis on the Spp1-related genes in HCC. **F**, **G** Correlation of SPP1 expression with the clinical characteristics of Grade and T stage in TCGA_LIHC. **H**–**K** Kaplan–Meier survival analysis show patients from TCGA_LIHC with high level of SPP1 expression had a significantly worse OS, PFS, DSS, and DFS. **L** Kaplan–Meier survival analysis of KLF2 from ICGC_LIRI dataset. **M** The ROC curve for SPP1 diagnosis

In addition, Kaplan–Meier survival analysis of subgroups presented those patients with higher KLFTs-17 expression achieved the worse OS (Fig. 5F). The clinical characteristics comparison analysis showed that the subgroup C1 was correlated to the more advanced T stage and higher histologic grade (Fig. 5G). The heatmap and volcano maps showed the differential gene expression (DEGs) between subgroups (Fig. 6H, I). Specifically, the functional enrichment analysis in Fig. 5J indicated that enriched terms were related to infection, cell proliferation, oncogenesis and so on. For GO analysis, the DEGs-up were highlighted in the entries “organelle fission”, “nuclear division”, “extracellular structure organization”, “extracellular matrix organization” and so on. However, the DEGs-down were mostly enriched in biological process related with metabolism. Taken together, the results above listed imply that highly expressed genes of subgroup classified by KLFTs-17 is more closely related to tumorigenesis and progression.

### Screening and identification of CAFs-related prognostic marker in HCC

To further derive promising markers associated with CAFs in HCC progression, we selected the C1 subgroup with the most striking features of KLFTs-17 to analyze the important role of CAF. COX regression analysis was applied, and SPP1 was identified as an independent prognostic factor associated with HCC fibrosis (Additional file 5: Table S4, Fig. 6A). Additionally, we depicted a nomogram to help predict the 1-, 3-, and 5-year survival of HCC patients (*P* < 0.001, C-index = 0.632, 95% CI 0.563–1) (Fig. 6B). The calibration curve in Fig. 6C showed a fine prognostic prediction performance of the nomogram established.

To further demonstrate the role of SPP1 in HCC, we explored the distribution of SPP1 expression across different types of tumor and normal tissues in TCGA. The result showed that SPP1 was increased not only in LIHC tumor tissue, but also in multiple cancers (Fig. 6D), such as BRCA, COAD, KIRP, LUAD, LUSC, READ and so on. To explore the molecular biological function of SPP1 in HCC, we used GSEA analysis to enrich the SPP1-related genes. Intriguingly, Fig. 6E revealed that SPP1 was involved not only in tumor stroma-related pathways, but also in the immune-related biological processes, such as “Allograft rejection” and “Inflammatory response”.

Furthermore, we analyzed the association between SPP1 expression and clinical characteristics. The results in Fig. 6F and G indicated a significant interaction of SPP1 expression with the tumor Grade and T stage. Additionally, we assessed the prognostic value of SPP1 in TCGA_LIHC and ICGC_LIRI. The KM curves presented HCC patients with higher level of SPP1 expression had a significantly worse OS, DSS, PFS, and RFS (Fig. 6H–K). What’s more, we noticed that SPP1, also known as osteopontin (OPN), had been reported to be a promising tumor marker for detecting metastatic disease in many tumors [38, 39]. Therefore, we assessed the diagnosis value of SPP1 in TCGA_LIHC, and the result of ROC curve showed SPP1 had a good sensitivity and high specificity for the diagnosis of HCC (AUC 0.732; CI 0.684–0.779) (Fig. 6L,M). The foregoing results support that patients with increased SPP1 expression have a poor prognosis. And SPP1 shows a favorable ability for HCC diagnosis.

### Relationship of KLF2 expression with immune infiltration in HCC

**Fig. 7:**
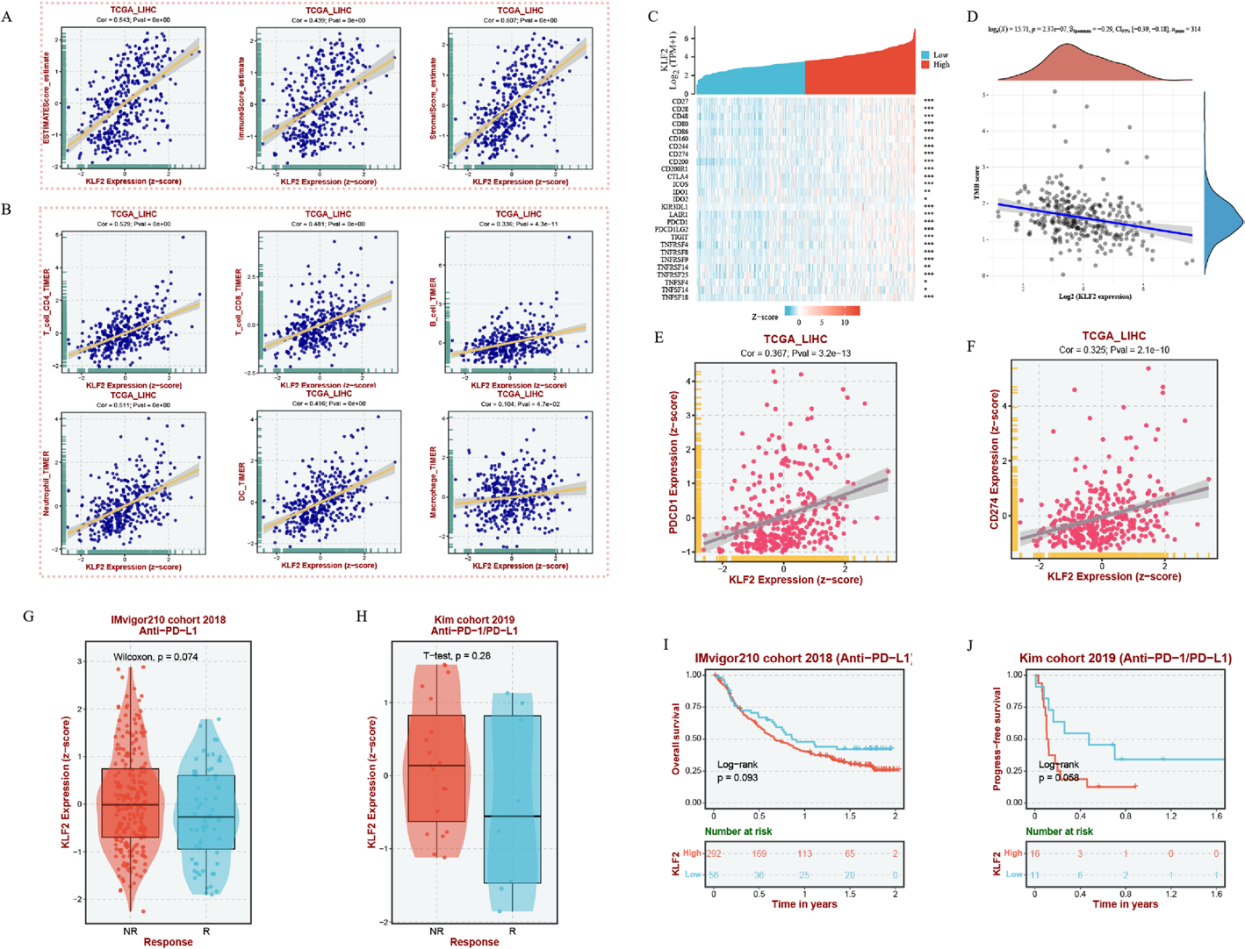
Multidimensional analysis between KLF2 expression and immune microenvironment in HCC. **A** KLF2 expression positively correlated with stromal score, immune score, and ESTIMATE score in HCC. **B** KLF2 expression is significantly positively related to infiltrating levels of CD4^+^ T cells, CD8^+^ T cells, B cells, dendritic cells, macrophages, and neutrophils in HCC. **C** Heat map of the correlation between the expression of KLF2 and immune-checkpoint-related genes. The different colors represent the trend of gene expression in different samples. **D** Correlation analysis between KLF2 gene expression and TMB. **E**, **F** Scatter plots show KLF2 expression is associated with PDCD1 and CD274. **G**, **H** Comparison of response to ICB treatments in KLF2 high- and low-expression groups. **I**, **J** Prognostic KM curves of high- and low-expression groups of KLF2. *P < 0.05, **P < 0.01, ***P < 0.001

The contribution of the tumor microenvironment to tumor prognosis cannot be negligible. ESTIMATE method was utilized to estimate non-tumor cell infiltration level involved in tumor microenvironment. The findings indicated that KLF2 expression was positively associated with Immune Score, Stromal Score and Estimate Score (Fig. 7A). Then we further investigated correlation of KLF2 expression with different types of immune cells. Scatter plots results showed a significantly positive correlation between KLF2 expression and immune cells infiltration in HCC based on the TIMER algorithm (Fig. 7B).

Immune checkpoint molecules expressed on the immune cells play a critical role in the immune responses and immunotherapy. Figure 7C showed the significant association between KLF2 and immune checkpoint molecules. Moreover, KLF2 expression was negatively correlated with tumor mutational burden (TMB) (ρ = − 0.29, *P* < 0.001) (Fig. 7D). Immune checkpoint inhibits (ICIs) therapy have dramatically improved outcomes of cancer patients in clinical practice, especially for PD-1 and PD-L1 (CD274). The scatter plots revealed the positive relationship of KLF2 with PD-1 and PD-L1 (CD274) (Fig. 7E, F). Then, we assessed the responsive of KLF2 in the immunotherapy cohorts, IMvigor210 cohort 2018 and Kim cohort 2019. Boxplots showed that patients with low KLF2-expressing level had a stronger response to these ICIs (Fig. 7G, H). Prognostic KM curves showed that patients with low KLF2 expression achieved better prognostic survival after ICIs treatments (Fig. 7I, J). Taken together, the above results suggest that KLF2 is highly correlated with the immune microenvironment in HCC, and advanced HCC patients with lower KLF2 expression derive greater benefit from ICIs treatments.

### Identification and analysis of a therapeutic biomarker CD3D

**Fig. 8:**
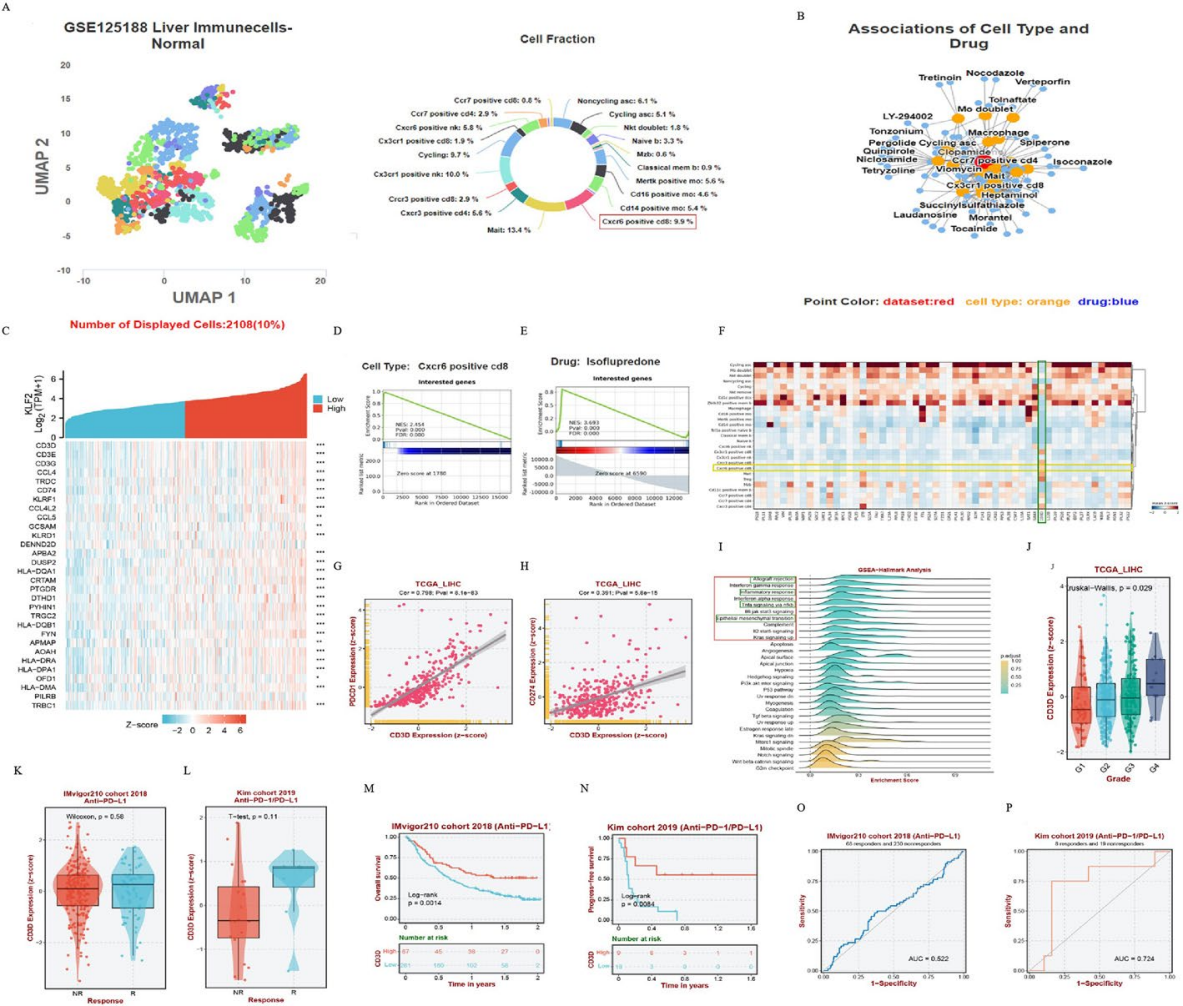
Comprehensive analysis of marker gene CD3D of CXCR6 CD8^+^ T cell. **A** UMAP plot and Cell Fraction plot show the cell type clustering and cell ratio of hepatic immune cells based on the single-cell sequencing analysis of GSE125188. **B** A network of the correlation between the cell type and drug response is also shown on the right (datasets are marked in red, cell types are marked in yellow, and drugs are marked in blue). **C** The heatmap shows association of KLF2 expression and marker genes of CXCR6 CD8^+^ T cell. **D**, **E** GSEA enrichment plot of CXCR6 CD8^+^-T-cell marker genes from GSE125188 scRNA dataset and differential genes expressed in CXCR6 CD8^+^-T-cell induced by the drug Isoflupredone. **F** Matrix plot of signature genes in GSE125188 dataset referring to CXCR6 CD8^+^ T cell and Isoflupredone. **G**, **H** Scatter plots show the correlation of CD3D with PDCD1 and CD274 (PDL1). **I** GSEA-Hallmark analysis on the CD3D-related genes in HCC. **J** The expression distribution of CD3D in the different grades of LIHC. **K**, **L** Comparison of response to ICB treatments in KLF2 high- and low-expression groups in IMvigor210 cohort 2018 and Kim cohort 2019. **M**, **N** Prognostic KM curves of high- and low-expression groups of CD3D in IMvigor210 2018 cohort and Kim cohort 2019. **O**, **P** ROC curves show the specificity and sensitivity of CD3D response in IMvigor210 cohort 2018 and Kim cohort 2019. *P < 0.05, **P < 0.01, ***P < 0.001

To better understand the association between KLF2 and heterogeneity of immune infiltration cells in liver tissue, we further analyzed the single-cell sequencing data of liver-resident immune cells derived from GSE125188 [40] using the CeDR Atlas database. As shown in Fig. 8A, the UMAP and the Cell Fraction plots showed the clustering of cell types and the proportion of cell types. The results showed that T cells accounted for the major proportion among all immune infiltration cells in the liver tissue. In addition, as Fig. 3E and F shown, KLF2 expression was higher in the T cells than in other immunocytes. In the anti-tumor immune response, CD8^+^ cytotoxic T lymphocytes (CTLs) played a main cellular effector role. Accordingly, we selected CXCR6 CD8^+^ T cells for further analysis. The heatmap in Fig. 8C indicated a strong association of KLF2 with the main marker genes expressed in CXCR6 CD8^+^ T cells [40]. As shown in Fig. 8B, a network of cell types and drug response was constructed, and for CXCR6 CD8^+^ T cells, the drug Isoflupredone showed the most significant statistical significance (Spearman cor: − 0.25: *P* value: 0.042). Then marker genes of CXCR6 CD8^+^ T cells and differential genes expressed by the drug Isoflupredone-induced in the CXCR6 CD8^+^ T cells were analyzed applying GSEA method (Fig. 8C–E). As Fig. 8F presented, the matrix plot visualized the main signature genes involved in associations between Isoflupredone and CXCR6 CD8^+^ T cells, with CD3D highlighted.

CD3D, an essential part of the T-cell receptor/CD3 complex (TCR/CD3 complex), is involved in development and signal transduction of T-cell [41]. Therefore, we further analyzed the association of CD3D with other TCRs. Scatter plots showed CD3D was statistically correlated with ICs, PDCD1 and CD274 (PDLD1) (Fig. 8G, H). Moreover, GSEA analysis was applied to enrich CD3D-related genes in HCC to better understand the molecular biological function of CD3D in HCC. Figure 8I showed the enriched entries were mainly immune-related biological processes. However, we noticed that the pathway “epithelial mesenchymal transition” was also included in the top ten items. Surprisingly, when comparing the enrichment results of SPP1-related genes and CD3D-related genes, we found that nearly half of the two pathways overlapped in the top ten enriched entries (Figs. 6E, 8I).

What’s more, Fig. 8J showed an essential distinction of CD3D expression in different Grades of HCC patients. And as tumor Grade advanced, CD3D expression levels were increasingly up-regulated. We noticed that the higher expression of CD3D was more responsive to ICIs (Fig. 8K, L). KM prognostic curves showed that higher CD3D expression predicted a better survival in HCC patients after ICIs treatments (Fig. 8M, N). Furthermore, area under curve (AUC) of ROC curves revealed CD3D had a moderate capacity to predicate HCC patients’ response to ICB therapy (Fig. 8O, P).

## Discussion

In our study, we focus on the exploration of KLF2, a predictive target gene of lnc-EPS15L1-2:1, which has been identified to have a strong association with HCC advancement in our previous research [5]. Surprisingly, we discovered that methylation, rather than genetic mutations is responsible for the down regulation of KLF2 expression in HCC. In addition, it has been reported that lncRNA ANRIL can regulate cell growth in vitro and in vivo through epigenetic silencing of KLF2 in HCC [42]. KM survival analysis reveals that HCC patients with decreased KLF2 expression tend to achieve a much worse OS, DSS, PFS, and RFS. This highlights the strong influence of KLF2 on the prognosis of HCC patients and its potential use as a dependable marker for prognostication.

KLF2, an important transcription factor, participates in many biological processes. The function enrichment results of the KLF2 targets, namely KLFTs, indicate that highly enriched entries focus on biological features associated with tumor stroma and immune responses. And we also assessed the differential levels of KLF2 expressed in cirrhosis tissue and HCC tissue. The findings further demonstrate that the expression level of KLF2 decreased continuously during the process of liver fibrosis to cirrhosis and then to hepatocarcinogenesis. Several studies have reported that KLF2 plays an important role in maintaining hepatic endothelial cell homeostasis and vascular integrity, and protects the liver from fibrosis or cirrhosis [43, 44]. TGF-β, a strong inducer of EMT, has been implicated as a key cytokine mediating liver fibrosis [45]. TGF-β is able to promote both fibrosis and carcinogenesis and shows increased levels in cirrhosis and advanced HCC [33]. KLF2 has been shown to function as a tumor suppressor through TGF-β/Smad signaling in HCC cells [46]. What’s more, other members of KLF family have been reported to significantly regulate the fibrotic process by transcriptionally regulating TGF-β expression, such as KLF4, KLF5, KLF6, and KLF15 [47–50], which indicates the great potential of KLF2 as a key gene involved in HCC fibrosis.

For most solid tumors, tumor matrix is an indispensable factor to promote tumor progression. Cancer associated fibroblasts (CAFs) are the predominant cells to improve tumor stromal microenvironment to facilitate tumor outgrowth. A growing number of studies have reported the essential contribution of CAFs to tumor progression [51, 52]. Most importantly, our study identifies a CAFs-related marker, SPP1, which is strongly associated with clinical characteristics and prognostic survival of HCC patients. Osteopontin (OPN), encoded by SPP1, has been implicated in multiple human diseases and has been shown to play an important regulatory role in HCC progression [53–55]. Therefore, we believe that the KLF2-SPP1 pathway is an important signaling axis that promotes liver tissue fibrosis, thus leading to HCC progression. However, whether SPP1 is a potential target of KLF2 involved in fibrosis deserves to be investigated experimentally.

OPN has been reported to be a promising tumor marker for detection in the diagnosis of many tumor progressions [38, 39]. And the ROC result reveals the high sensitivity and specificity of SPP1 diagnosis value in HCC, indicting the strong capacity of SPP1 as a prospective biomarker for future diagnosis and prognosis prediction for HCC patients.

Multidimensional analyses of the correlation between KLF2 and immune infiltration showed an essential involvement of KLF2 in regulating tumor immune microenvironment. We found that patients with low KLF2 expression levels were more responsive to ICIs therapy and achieved a better prognosis and survival, indicating that advanced HCC patients with lower KLF2 expression levels are more suitable for ICIs therapy.

The results of single-cell level expression analysis from multiple platforms reveal the expression distribution of KLF2 is highly distinct in different cells of liver tissue. High levels of expression in immune cells (especially T cells), endothelial cells, and fibroblasts, but low levels in hepatocytes. Kuo et al. have demonstrated that KLF2 is expressed in both CD4^+^ and CD8^+^ T cells. The expression of KLF2 mRNA and protein is significantly down-regulated upon activation of resting T cells via T cell receptor (TCR) [56], which suggests an essential role of KLF2 on T cell function. And KLF2 has also been shown to inhibit the proliferation and growth of Jurkat T leukemia cells [57, 58]. These existed researches have suggested that KLF2 plays a critical role in maintaining the function of T cells. CD8^+^ T cells performs important functions in the immune response, and cluster analysis shows CXCR6 CD8^+^ T cell is the predominant T cell type in liver tissue. And KLF2 is significantly associated with biomarkers of CXCR6 CD8^+^ T cell. In addition, we found that CD3D, an important membrane protein for CXCR6 CD8^+^ T cells to exert immune responsive, was associated with HCC progression and immunotherapy. Furthermore, many studies have reported CD3D as a promising prognostic and therapeutic biomarker [59–61]. These studies further confirmed our findings that CD3D is prospective to be an emerging marker for HCC immunotherapy. Therefore, we speculate CD3D is a key mediator of KLF2 involvement in HCC immune response.

## Conclusion

Our study identifies the important function of KLF2 for advanced HCC by affecting the fibrosis and immune infiltration, and provides new perspectives on exploring the molecular mechanism for HCC advancement, emphasizing the potential of KLF2 as a new biomarker for improving the prognosis of advanced HCC patients in clinical practice.

### Supplementary Information

**Additional file 1: Fig. S1.** The expression distribution of KLF family members and regulators of NOS enzymes in tumor tissues and normal tissues of TCGA-LIHC and ICGC-LIRI. **Fig. S2.** The prognostic survival value of KLF family members and regulators of NOS enzymes from TCGA-LIHC and ICGC-LIRI.** Fig. S3.** Analysis of the relationship between EMT-markers and KLF2 and the expression distribution of EMT-markers in the GSE25097. **Fig. S4.** Forest plots presentation of univariate Cox regression of KLFTs. Table S1: Target genes of the KLF2 from the CHEA Transcription Factor Targets dataset in Harmonizome platform, namely KLFTs.

**Additional file 2: Table S1.** Target genes of the KLF2 from the CHEA Transcription Factor Targets dataset in Harmonizome platform, namely KLFTs.

**Additional file 3: Table S2.** The co-expression analysis of KLF2 and KLFTs.

**Additional file 4: Table S3.** The common CAFs-related marker genes by consulting relevant references.

**Additional file 5: Table S4.** Univariate COX regression analysis of CAFs in the C1 subgroup.

## Data Availability

The data of this study are available in the The Cancer Genome Atlas (TCGA, https://portal.gdc.cancer.gov), the International Cancer Genome Consortium database (ICGC, https://daco.icgc.org/), the Gene Expression Comprehensive Database (GEO, http://www.ncbi.nlm.nih.gov/geo), cBioPortal platform (https://www.cbioportal.org/), CeDR Atlas platform (https://ngdc.cncb.ac.cn/cedr/), the Human Protein Atlas (HPA, https://www.proteinatlas.org/), and Harmonizome platform (https://maayanlab.cloud/Harmonizome/).

## Abbreviations

HCC: Hepatocellular carcinoma
KLF: Kruppel-like factor
DEGs: Differentially expressed genes
TCGA: The Cancer Genome Atlas
GEO: The gene expression comprehensive database
ICGC: International cancer genome consortium
HPA: Human Protein Atlas
OS: Overall survival
RFS: Relapse free survival
PFS: Progression free survival
DFS: Disease free survival
HR: Hazard ratio
BRCA: Breast invasive carcinoma
COAD: Colon adenocarcinoma
KIRP: Kidney renal papillary cell carcinoma
LUAD: Lung adenocarcinoma
LUSC: Lung squamous cell carcinoma
READ: Rectum adenocarcinoma
KLFTs: KLF2 targets
CAFs: Cancer associated fibroblasts
ROC: Receiver operating characteristic
AUC: Area under curve
ECM: Extracellular matrix
GSEA: Gene set enrichment analysis
KEGG: Kyoto Encyclopedia of Genes and Genomes
SPP1: Secreted phosphoprotein 1
OPN: Osteopontin
TCR: T cell receptor
TMB: Tumor mutational burden
ICIs: Immune checkpoint inhibits
