# Correction: Comprehensive analysis of KLF2 as a prognostic biomarker associated with fibrosis and immune infiltration in advanced hepatocellular carcinoma

**DOI:** 10.1186/s12859-023-05623-3

**Published:** 2024-01-03

**Authors:** Xue-Qin Chen, Jie Ma, Di Xu, Zuo-Lin Xiang

**Affiliations:** 1 Department of Radiation Oncology, Shanghai East Hospital, School of Medicine, Tongji University, Shanghai, 200120 China; 2 Department of Radiation Oncology, Shanghai East Hospital Ji’an Hospital, Jiangxi, 343000 China

**Correction : BMC Bioinformatics (2023) 24:270** 10.1186/s12859-023-05391-0

Following the publication of the original article [1], the authors identified that the funding statement was incorrect. The correct Funding is given below.

The incorrect funding is: This work was supported by the National Natural Science Foundation of China (Grant No. 81960525 and 82160591), Science and the Technology Commission of Shanghai Municipality (Grant No. 19DZ1930900), The Top-level Clinical Discipline Project of Shanghai Pudong (PWYgf2021-07), Key Project of Clinical Research of Shanghai East Hospital, Tongji University (Grant No. DFLC2022012) and Key Specialty Construction Project of Shanghai Pudong New Area Health Commission (Grant No. PWZzk2022-02).

The correct funding is: This work was supported by the National Natural Science Foundation of China (Grant No. 81960525 and 82160591), Science and the Technology Commission of Shanghai Municipality (Grant No. 19DZ1930903), The Top-level Clinical Discipline Project of Shanghai Pudong (PWYgf2021-07), Key Project of Clinical Research of Shanghai East Hospital, Tongji University (Grant No. DFLC2022012) and Key Specialty Construction Project of Shanghai Pudong New Area Health Commission (Grant No. PWZzk2022-02).

The original article [1] has been corrected.
