# Supplementary material for: Comprehensive analysis of KLF2 as a prognostic biomarker associated with fibrosis and immune infiltration in advanced hepatocellular carcinoma
Source: BMC Bioinformatics. 2023 Jun 29;24:270. doi: 10.1186/s12859-023-05391-0 (PMC10308631; doi:10.1186/s12859-023-05391-0)
Supplement: Supplementary file 1 — Additional file 1: Fig. S1. The expression distribution of KLF family members and regulators of NOS enzymes in tumor tissues and normal tissues of TCGA-LIHC and ICGC-LIRI. Fig. S2. The prognostic survival value of KLF family members and regulators of NOS enzymes from TCGA-LIHC and ICGC-LIRI. Fig. S3. Analysis of the relationship between EMT-markers and KLF2 and the expression distribution of EMT-markers in the GSE25097. Fig. S4. Forest plots presentation of univariate Cox regression of KLFTs. Table S1: Target genes of the KLF2 from the CHEA Transcription Factor Targets dataset in Harmonizome platform, namely KLFTs. [file 12859_2023_5391_MOESM1_ESM.docx]

**Additional file 1**

**Legends of Supplementary Figures**


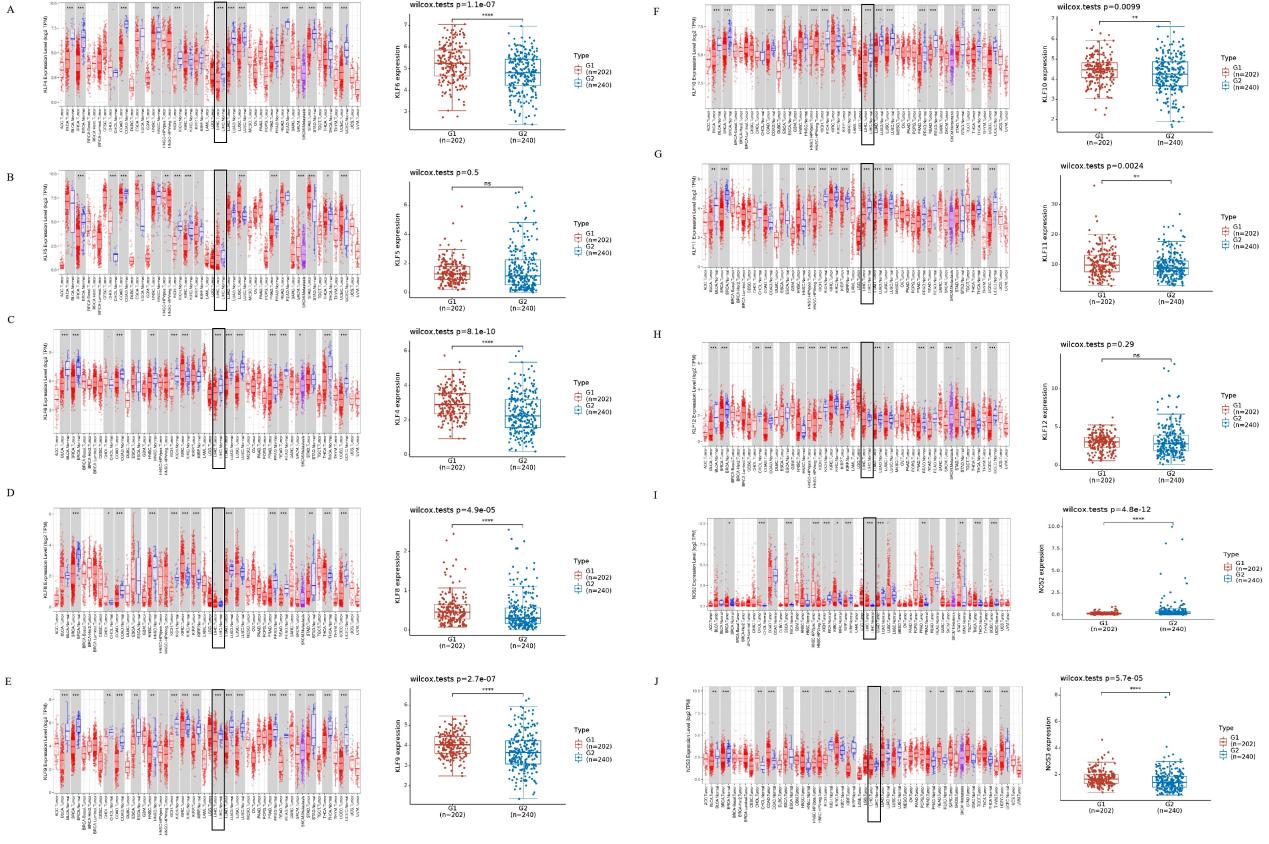


Supplementary Fig1. The expression distribution of KLF family members and regulators of NOS enzymes in tumor tissues and normal tissues of TCGA-LIHC and ICGC-LIRI.


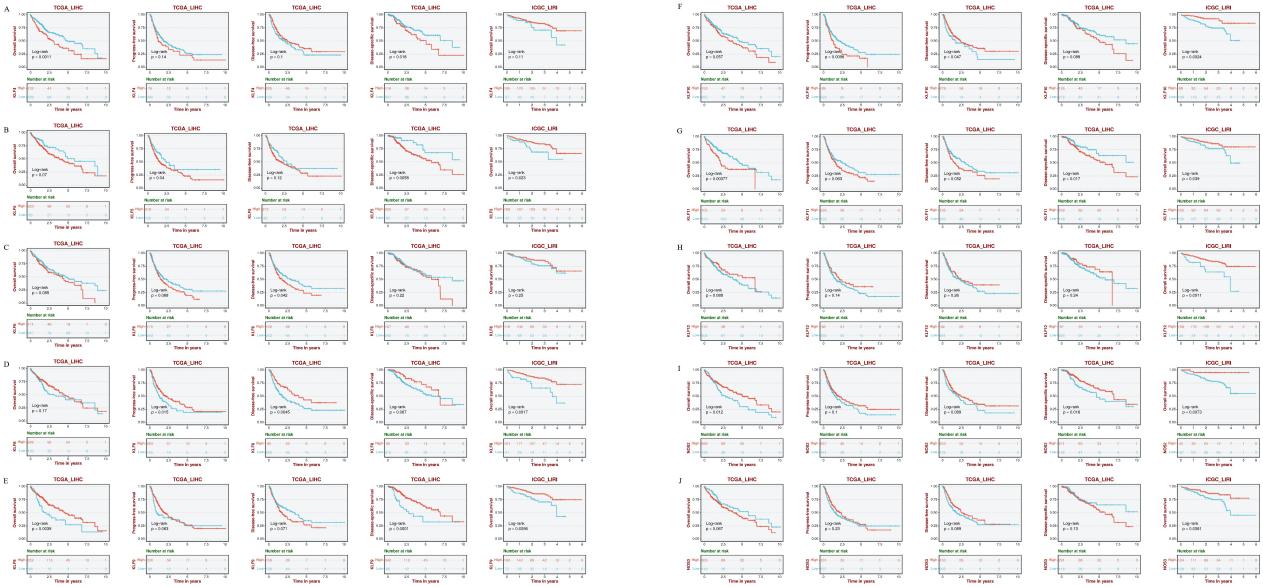


Supplementary Fig 2. The prognostic survival value of KLF family members and regulators of NOS enzymes from TCGA-LIHC and ICGC-LIRI.


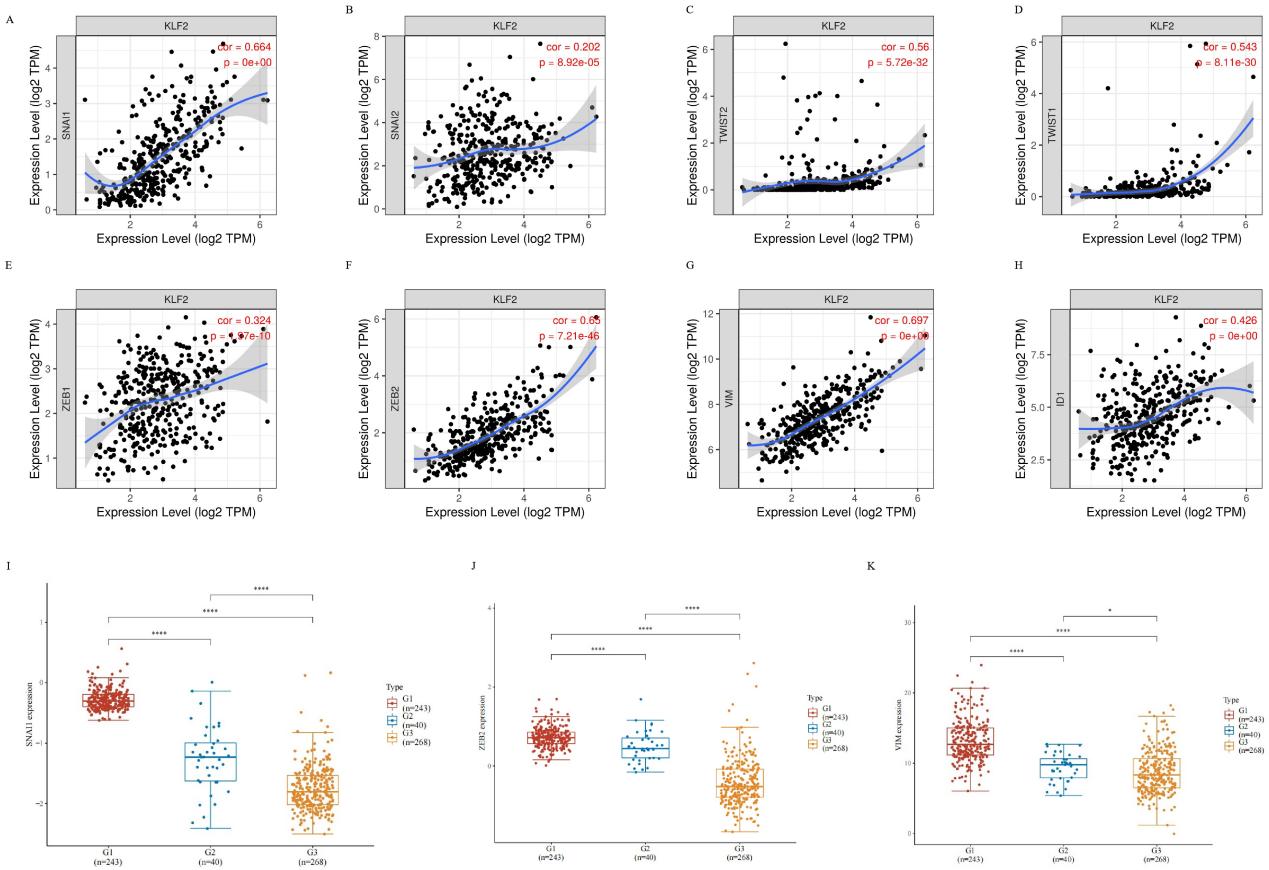


Supplementary Fig 3. Analysis of the relationship between EMT-markers and KLF2 and the expression distribution of EMT-markers in the GSE25097.


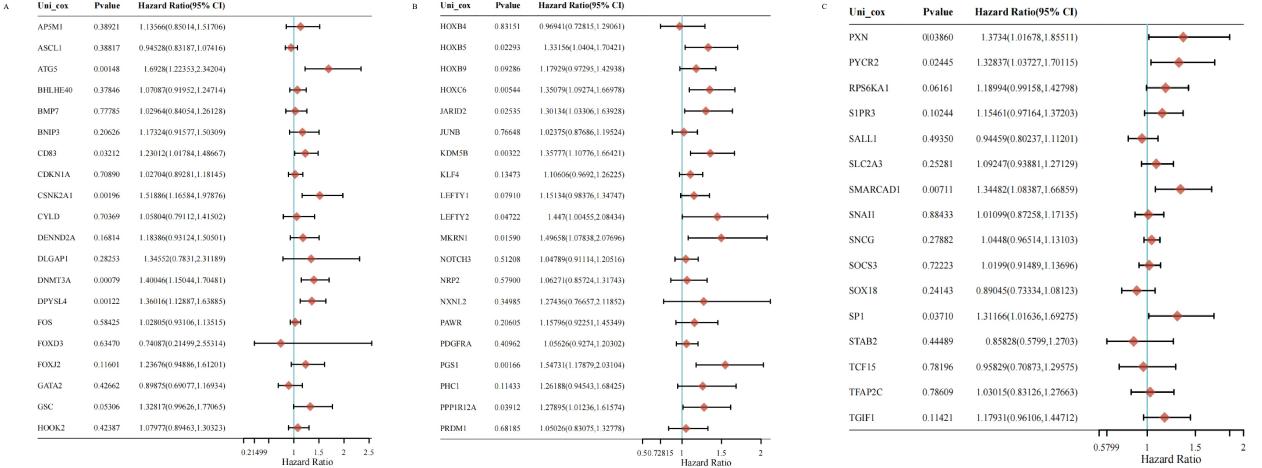


Supplementary Fig 4. Forest plots presentation of univariate Cox regression of KLFTs. Table S1: Target genes of the KLF2 from the CHEA Transcription Factor Targets dataset in Harmonizome platform, namely KLFTs.
