# Supplementary material for: Comprehensive analysis of KLF2 as a prognostic biomarker associated with fibrosis and immune infiltration in advanced hepatocellular carcinoma
Source: BMC Bioinformatics. 2023 Jun 29;24:270. doi: 10.1186/s12859-023-05391-0 (PMC10308631; doi:10.1186/s12859-023-05391-0)
Supplement: Supplementary file 2 — Additional file 2: Table S1. Target genes of the KLF2 from the CHEA Transcription Factor Targets dataset in Harmonizome platform, namely KLFTs. [file 12859_2023_5391_MOESM2_ESM.docx]

**Additional file 2**

**Supplementary Table 1.**

Target genes of the KLF2 from the CHEA Transcription Factor Targets dataset in Harmonizome platform, namely KLFTs.

| **KTFTs** | |
| --- | --- |
| **source** | **source_id** |
| DLGAP1 | 9229 |
| DTNB | 1838 |
| BHLHE40 | 8553 |
| RPS6KA1 | 6195 |
| PXN | 5829 |
| UBE2V1 | 7335 |
| MSRA | 4482 |
| TEX14 | 56155 |
| CYLD | 1540 |
| RYBP | 23429 |
| FOXJ2 | 55810 |
| KLF4 | 9314 |
| FOXD3 | 27022 |
| GSC | 145258 |
| LEFTY2 | 7044 |
| DPYSL4 | 10570 |
| BMP7 | 655 |
| HOXB5 | 3215 |
| HOXB9 | 3219 |
| HOXC8 | 3224 |
| HOXB4 | 3214 |
| HOXB13 | 10481 |
| SIX1 | 6495 |
| HOXC6 | 3223 |
| ASCL1 | 429 |
| MFSD2A | 84879 |
| SOX18 | 54345 |
| NRP2 | 8828 |
| SALL1 | 6299 |
| TFAP2C | 7022 |
| BMP4 | 652 |
| GATA2 | 2624 |
| CD83 | 9308 |
| MYCL | 4610 |
| PDGFRA | 5156 |
| GPR101 | 83550 |
| PHC1 | 1911 |
| SOX2 | 6657 |
| SLC2A3 | 6515 |
| NANOG | 79923 |
| LEFTY1 | 10637 |
| TCF15 | 6939 |
| PRDM1 | 639 |
| DENND2A | 27147 |
| S1PR3 | 1903 |
| ATG5 | 9474 |
| SMARCAD1 | 56916 |
| RAD51C | 5889 |
| SP1 | 6667 |
| RAD23B | 5887 |
| RRP1B | 23076 |
| KDM5B | 10765 |
| PAWR | 5074 |
| JARID2 | 3720 |
| KLF9 | 687 |
| SOCS3 | 9021 |
| CDKN1A | 1026 |
| TGIF1 | 7050 |
| KANK1 | 23189 |
| MKRN1 | 23608 |
| JUNB | 3726 |
| AP1M1 | 8907 |
| FOS | 2353 |
| CSNK2A1 | 1457 |
| TRPM3 | 80036 |
| DNMT3A | 1788 |
| ARL8B | 55207 |
| ATG4C | 84938 |
| PGS1 | 9489 |
| HOOK2 | 29911 |
| RPN1 | 6184 |
| PPP1R12A | 4659 |
| STAB2 | 55576 |
| BNIP3 | 664 |
| TCEA2 | 6919 |
| RABIF | 5877 |
| SNAI1 | 6615 |
| SNCG | 6623 |
| CNIH1 | 10175 |
| DNAJC19 | 131118 |
| PYCR2 | 29920 |
| PLA2G1B | 5319 |
| SIX4 | 51804 |
| AP5M1 | 55745 |
| TPT1 | 7178 |
| SERPINA3 | 12 |
| NOTCH3 | 4854 |
| NXNL2 | 158046 |
| SP7 | 121340 |
| ZMYM3 | 9203 |
| GJB1 | 2705 |
| ATOH1 | 474 |
| DMRT1 | 1761 |
| GSX2 | 170825 |
| OTX2 | 5015 |
| DPPA3 | 359787 |
| ZIC3 | 7547 |
| KLF2 | 10365 |
