# Supplementary material for: Comprehensive analysis of KLF2 as a prognostic biomarker associated with fibrosis and immune infiltration in advanced hepatocellular carcinoma
Source: BMC Bioinformatics. 2023 Jun 29;24:270. doi: 10.1186/s12859-023-05391-0 (PMC10308631; doi:10.1186/s12859-023-05391-0)
Supplement: Supplementary file 3 — Additional file 3: Table S2. The co-expression analysis of KLF2 and KLFTs. [file 12859_2023_5391_MOESM3_ESM.docx]

**Additional file 3**

**Supplementary Table 2.**

The co-expression analysis of KLF2 and KLFTs.

| **Gene1** | **Gene2** | **cor** | ***P*.value** | ***P* star** |
| --- | --- | --- | --- | --- |
| KLF2 | AP5M1 | 0.164841845 | 0.001441612 | ** |
| KLF2 | ASCL1 | -0.136947456 | 0.008257669 | ** |
| KLF2 | ATG5 | 0.180783575 | 0.000475821 | ** |
| KLF2 | BHLHE40 | 0.127395503 | 0.014115076 | * |
| KLF2 | BMP7 | 0.296386337 | 5.86E-09 | ** |
| KLF2 | BNIP3 | -0.272769465 | 1.06E-07 | ** |
| KLF2 | CD83 | 0.318509319 | 4.29E-10 | ** |
| KLF2 | CDKN1A | 0.165428623 | 0.001403516 | ** |
| KLF2 | CSNK2A1 | 0.13819505 | 0.007684105 | ** |
| KLF2 | CYLD | 0.302072693 | 2.89E-09 | ** |
| KLF2 | DENND2A | 0.550238907 | 9.52E-31 | ** |
| KLF2 | DLGAP1 | 0.349621477 | 4.17E-12 | ** |
| KLF2 | DNMT3A | 0.139822389 | 0.007029224 | ** |
| KLF2 | DPYSL4 | 0.35892632 | 1.01E-12 | ** |
| KLF2 | FOS | 0.484837746 | 0 | ** |
| KLF2 | FOXD3 | 0.109386195 | 0.035192124 | * |
| KLF2 | FOXJ2 | 0.277142662 | 5.74E-08 | ** |
| KLF2 | GATA2 | 0.594822399 | 7.12E-37 | ** |
| KLF2 | GSC | 0.228162892 | 9.06E-06 | ** |
| KLF2 | HOOK2 | 0.201247612 | 9.49E-05 | ** |
| KLF2 | HOXB4 | 0.713228663 | 6.58E-59 | ** |
| KLF2 | HOXB5 | 0.452408593 | 4.06E-20 | ** |
| KLF2 | HOXB9 | 0.22934788 | 8.11E-06 | ** |
| KLF2 | HOXC6 | 0.182019711 | 0.000425734 | ** |
| KLF2 | JARID2 | 0.108952923 | 0.035970542 | * |
| KLF2 | JUNB | 0.486111666 | 0 | ** |
| KLF2 | KDM5B | 0.214821508 | 3.14E-05 | ** |
| KLF2 | KLF4 | 0.353985905 | 2.96E-12 | ** |
| KLF2 | LEFTY1 | 0.223716878 | 1.36E-05 | ** |
| KLF2 | LEFTY2 | 0.341046204 | 1.48E-11 | ** |
| KLF2 | MKRN1 | 0.129049883 | 0.012904396 | * |
| KLF2 | NOTCH3 | 0.551775521 | 0 | ** |
| KLF2 | NRP2 | 0.61968924 | 0 | ** |
| KLF2 | NXNL2 | 0.439114531 | 6.41E-19 | ** |
| KLF2 | PAWR | 0.163687666 | 0.00155846 | ** |
| KLF2 | PDGFRA | 0.585505972 | 1.63E-35 | ** |
| KLF2 | PGS1 | 0.126205484 | 0.01499832 | * |
| KLF2 | PHC1 | 0.408654362 | 2.29E-16 | ** |
| KLF2 | PPP1R12A | 0.288753303 | 1.48E-08 | ** |
| KLF2 | PRDM1 | 0.565415933 | 0 | ** |
| KLF2 | PXN | 0.231121044 | 6.87E-06 | ** |
| KLF2 | PYCR2 | 0.121435269 | 0.019344364 | * |
| KLF2 | RPS6KA1 | 0.143666012 | 0.005566855 | ** |
| KLF2 | S1PR3 | 0.333623183 | 4.27E-11 | ** |
| KLF2 | SALL1 | -0.110940183 | 0.032659342 | * |
| KLF2 | SLC2A3 | 0.44645042 | 1.42E-19 | ** |
| KLF2 | SMARCAD1 | 0.193800539 | 0.000177415 | ** |
| KLF2 | SNAI1 | 0.647545807 | 0 | ** |
| KLF2 | SNCG | 0.328133394 | 1.19E-10 | ** |
| KLF2 | SOCS3 | 0.396669385 | 9.13E-16 | ** |
| KLF2 | SOX18 | 0.532056672 | 0 | ** |
| KLF2 | SP1 | 0.192818956 | 0.000191547 | ** |
| KLF2 | STAB2 | 0.487678549 | 1.46E-23 | ** |
| KLF2 | TCF15 | 0.217867437 | 2.30E-05 | ** |
| KLF2 | TFAP2C | 0.321242195 | 2.37E-10 | ** |
| KLF2 | TGIF1 | 0.119772664 | 0.021075567 | * |
