# Supplementary material for: Comprehensive analysis of KLF2 as a prognostic biomarker associated with fibrosis and immune infiltration in advanced hepatocellular carcinoma
Source: BMC Bioinformatics. 2023 Jun 29;24:270. doi: 10.1186/s12859-023-05391-0 (PMC10308631; doi:10.1186/s12859-023-05391-0)
Supplement: Supplementary file 4 — Additional file 4: Table S3. The common CAFs-related marker genes by consulting relevant references. [file 12859_2023_5391_MOESM4_ESM.docx]

**Additional file 4**

**Supplementary Table 3.**

The common CAFs-related marker genes by consulting relevant references.

| **CAFs-related marker genes** |
| --- |
| TNC |
| LAMA5 |
| ITGB1 |
| ANGPT1 |
| ANGPT2 |
| CXCL12 |
| STX2 |
| TGFB1 |
| HGF |
| EREG |
| SPP1 |
| POSTN |
| CSPG4 |
| PDPN |
| MFAP5 |
| FGF5 |
| CXCL5 |
| MMP1 |
| IGFL2 |
| ADAM32 |
| FGF8 |
| FGF17 |
| FGF19 |
| FGF4 |
| FGF23 |
| ACTA2 |
| ATL1 |
| FAP |
| VIM |
| DES |
| DDR2 |
| PDGFRA |
| PDGFRB |
