# Supplementary material for: Comprehensive analysis of KLF2 as a prognostic biomarker associated with fibrosis and immune infiltration in advanced hepatocellular carcinoma
Source: BMC Bioinformatics. 2023 Jun 29;24:270. doi: 10.1186/s12859-023-05391-0 (PMC10308631; doi:10.1186/s12859-023-05391-0)
Supplement: Supplementary file 5 — Additional file 5: Table S4. Univariate COX regression analysis of CAFs in the C1 subgroup. [file 12859_2023_5391_MOESM5_ESM.docx]

**Additional file 5**

**Supplementary Table 4.**

Univariate COX regression analysis of CAFs in the C1 subgroup.

| **Characteristics** | **Total(N)** | **Univariate analysis** | |
| --- | --- | --- | --- |
|  |  | **Hazard ratio (95% CI)** | **P value** |
| TNC | 193 |  |  |
| Low | 96 |  |  |
| High | 97 | 1.021(0.878-1.186) | 0.790 |
| LAMA5 | 193 |  |  |
| Low | 96 |  |  |
| High | 97 | 1.108(0.919-1.336) | 0.281 |
| ITGB1 | 193 |  |  |
| Low | 96 |  |  |
| High | 97 | 1.062 (0.830-1.359) | 0.634 |
| **ANGPT1** | 193 |  |  |
| Low | 96 |  |  |
| High | 97 | 1.416 (1.076-1.865) | **0.013** |
| **ANGPT2** | 193 |  |  |
| Low | 96 |  |  |
| High | 97 | 1.421 (1.106-1.824) | **0.006** |
| CXCL12 | 193 |  |  |
| Low | 96 |  |  |
| High | 97 | 0.950 (0.825-1.095) | 0.482 |
| STX2 | 193 |  |  |
| Low | 96 |  |  |
| High | 97 | 1.123 (0.830-1.521) | 0.453 |
| TGFB1 | 193 |  |  |
| Low | 96 |  |  |
| High | 97 | 1.115 (0.955-1.302) | 0.169 |
| HGF | 193 |  |  |
| Low | 96 |  |  |
| High | 97 | 0.988(0.818-1.193) | 0.899 |
| EREG | 193 |  |  |
| Low | 96 |  |  |
| High | 97 | 1.063 (0.866-1.305) | 0.556 |
| **SPP1** | 193 |  |  |
| Low | 96 |  |  |
| High | 97 | 1.131 (1.054-1.521) | **<0.001** |
| POSTN | 193 |  |  |
| Low | 96 |  |  |
| High | 97 | 1.041 (0.929-1.165) | 0.491 |
| CSPG4 | 193 |  |  |
| Low | 96 |  |  |
| High | 97 | 1.156 (0.906-1.476) | 0.244 |
| PDPN | 193 |  |  |
| Low | 96 |  |  |
| High | 97 | 1.017 (0.821-1.258) | 0.880 |
| MFAP5 | 193 |  |  |
| Low | 96 |  |  |
| High | 97 | 0.842(0.511-1.386) | 0.499 |
| CXCL5 | 193 |  |  |
| Low | 96 |  |  |
| High | 97 | 1.083 (0.987-1.188) | 0.093 |
| **MMP1** | 193 |  |  |
| Low | 96 |  |  |
| High | 97 | 1.317 (1.147-1.512) | **<0.001** |
| IGFL2 | 193 |  |  |
| Low | 96 |  |  |
| High | 97 | 1.372 (0.885-2.128) | 0.157 |
| ADAM32 | 193 |  |  |
| Low | 96 |  |  |
| High | 97 | 1.156 (0.646-2.067) | 0.625 |
| ACTA2 | 193 |  |  |
| Low | 96 |  |  |
| High | 97 | 0.890(0.734-1.080) | 0.238 |
| ATL1 | 193 |  |  |
| Low | 96 |  |  |
| High | 97 | 0.650(0.321-1.317) | 0.232 |
| DDR2 | 193 |  |  |
| Low | 96 |  |  |
| High | 97 | 0.906 (0.696-1.179) | 0.462 |
| DES | 193 |  |  |
| Low | 96 |  |  |
| High | 97 | 0.842(0.511-1.386) | 0.499 |
| FAP | 193 |  |  |
| Low | 96 |  |  |
| High | 97 | 0.966 (0.745-1.254) | 0.797 |
| FGF17 | 193 |  |  |
| Low | 96 |  |  |
| High | 97 | 1.013 (0.567-1.810) | 0.965 |
| FGF19 | 193 |  |  |
| Low | 96 |  |  |
| High | 97 | 0.842(0.511-1.386) | 0.499 |
| FGF23 | 193 |  |  |
| Low | 96 |  |  |
| High | 97 | 0.708 (0.178-2.821) | 0.625 |
| FGF4 | 193 |  |  |
| Low | 96 |  |  |
| High | 97 | 0.814 (0.486-1.362) | 0.432 |
| FGF8 | 193 |  |  |
| Low | 96 |  |  |
| High | 97 | 1.010(0.682-1.500) | 0.962 |
| PDGFRA | 193 |  |  |
| Low | 96 |  |  |
| High | 97 | 0.984 (0.845-1.146) | 0.835 |
| PDGFRB | 193 |  |  |
| Low | 96 |  |  |
| High | 97 | 0.937 (0.759-1.156) | 0.541 |
| VIM | 193 |  |  |
| Low | 96 |  |  |
| High | 97 | 1.108(0.889-1.380) | 0.363 |
| FGF5 | 193 |  |  |
| Low | 96 |  |  |
| High | 97 | 1.139 (0.673-1.928) | 0.627 |
